# Intrauterine Hypoxia Changed the Colonization of the Gut Microbiota in Newborn Rats

**DOI:** 10.3389/fped.2021.675022

**Published:** 2021-04-26

**Authors:** Yan Sun, Lei Li, Jiayu Song, Wei Mao, Kaihao Xiao, Chunming Jiang

**Affiliations:** Department of Neonatology, The First Affiliated Hospital of Harbin Medical University, Harbin, China

**Keywords:** intrauterine hypoxia, gut microbiota, high-throughput sequencing, newborn rats, colonization, diversity

## Abstract

**Background:** Accumulating evidence suggests a connection between the gut microbiota and neonatal diseases. Hypoxia may play an important role in the intestinal lesions in neonates.

**Objective:** This study aims to determine whether the gut microbiota differs between intrauterine hypoxic rats and healthy controls and to identify the factors that influence the changes in the gut microbiota.

**Methods:** We constructed an intrauterine hypoxia model in rats and collected the intestinal contents of intrauterine hypoxic newborn rats and normal newborn rats within 4 h and on the seventh day after birth. They were divided them into the intrauterine hypoxia first-day group (INH1), intrauterine hypoxia seventh-day group (INH7), normal first-day group (NOR1), and normal seventh-day group (NOR7). The contents of the intestines were sequenced with 16S rRNA sequencing, the sequencing results were analyzed for biological information, and the differences in the diversity, richness, and individual taxa among the groups were analyzed.

**Results:** The abundance of the gut microbiota of neonatal rats with intrauterine hypoxia was higher than that of the control group rats. Intrauterine hypoxia altered the structural composition of the gut microbiota in neonatal rats. The INH1 group showed increased species richness, phylogenetic diversity, and β-diversity, and altered relative abundance in several taxa compared to those in the control group. The differences in the microbiota among the four groups were significantly higher than those within the group, and the differences in the abundance and diversity of the INH7 and NOR7 groups decreased after 7 days of suckling. Functional analysis based on the Cluster of Orthologous Groups (COG) suggested that 23 functional COG categories. There was no significant difference in the functional categories between the hypoxia group and the normal group.

**Conclusion:** Intrauterine hypoxia changed the initial colonization of the gut microbiota in neonatal rats. It could increase the species richness and β-diversity of the gut microbiota, and altered relative abundances of several taxa.

## Introduction

Intrauterine hypoxia is a prevalent problem in fetuses and neonates, and it is one of the important causes leading to fetal intrauterine distress, growth restriction, hypoxic-ischemic encephalopathy of neonates, and even fetal and neonatal death. During birth and thereafter, microbes colonize the gastrointestinal tract and forms a relatively stable microbial population (1–3). Due to the redistribution of blood in the body, the intestinal microenvironment of neonates with intrauterine hypoxia changes, resulting in an imbalance of the gut microbiota and subsequently in the production of a large number of toxic metabolites and an induced inflammatory response. Newborns are susceptible to immune dysfunction, and gut microbiota dysfunction may increase the risk of related diseases in adulthood.

Necrotizing enterocolitis (NEC) is a catastrophic disease that is a major cause of mortality in preterm infants who survive the first few days after birth (4). Necrotizing enterocolitis occurs in 7% of infants born at <1,500 g and up to 5% of those admitted to the neonatal intensive care unit (5–8). Necrotizing enterocolitis is associated with high mortality (15–30%) and long-term neurodevelopmental morbidity (5, 9). Premature birth, formula feeding, abnormal bacterial colonization, and intestinal barrier dysfunction have been identified as the main etiological factors that substantially potentiate the risk of NEC (4). Among these, the damage to the intestinal barrier is considered to be the most predominant cause of NEC in neonates. Maintaining the integrity of the intestinal barrier helps protect against NEC (10). Hypoxia is one of the factors known to damage the intestinal barrier (11). As one of the major pathological factors for infant intestines suffering from NEC (12), hypoxia induces cell death, and deterioration of the integrity of the intestinal barrier, thereby reducing the restorative ability of crypt stem cells (13). In addition, hypoxia can sensitize the immune response in the gut, and aggravates inflammatory reactions of the mucosal barrier to the bacterial invasion (14). In fact, the excessive inflammatory response represents a major pathogenesis of NEC in preterm infants (15).

Intestinal microecology is the most complex microecosystem in the human body. It not only is involved in the digestion of food, the absorption of nutrients, and the synthesis of vitamins but also plays an important role in immune regulation (16). Gut microbiota dysbiosis is closely associated with localized gastroenterologic disorders (17). It can even further affect the normal development of the nervous system and respiratory system through the brain–gut axis and lung–gut axis (18, 19), especially in the neonatal period. In early life, the gut microbiota is dynamic and its development is susceptible to host and environmental factors (20). Host–microbe interactions influence the intestinal tract and immune system (21). Hypoxia is a potential influencer of microbiota development, and hypoxia might interfere with microbiota development, especially with the colonization of anaerobic bacteria. However, there are limited studies on whether intrauterine hypoxia induces NEC through the disturbance of the gut microbiota. Therefore, additional studies are needed to clarify the relationship between intrauterine hypoxia and the gut microbiota.

## Materials and Methods

### Animals Model

All procedures strictly adhered to the National Institutes of Research guidelines on the ethical use of animals and were under license from the local ethics review committee of First Affiliated Harbin Medical University. Wistar rats were supplied by the Animal Experiment Center of Harbin Medical University and were raised in a clean-grade experimental environment. Animals were housed under a 12-h light/dark cycle (22 ± 2°C) and fed with a standard chow diet and water. The cages and water bottles were sterilized every day. All endeavors were made to minimize the sufferings of the animals involved in the experiment. Female and male Wistar rats at the age of 3 months were mated in cages at a ratio male-to-female ratio of 1:2. The female rats were judged to be pregnant by checking whether there was a pregnancy plug in the vagina or whether there were sperm in a vaginal secretion smear of the female rats obtained the next morning, which was taken as the 0th day of pregnancy. The pregnant rats were kept in a room with a temperature of 21°C, a humidity of 60%, and 12-h light and dark cycle. One group of rats was stimulated with hypoxia after conception to construct an intrauterine hypoxia model as the experimental group, and the other group was treated as the control group without any intervention after conception. Intestinal contents of neonates with intrauterine hypoxia and normal neonates within 24 h and 7 days after birth were collected, respectively, and were divided into four groups: the first-day of intrauterine hypoxia group (INH1), the seventh-day of intrauterine hypoxia group (INH7), the first-day of normal group (NOR1), and the seventh-day of normal group (NOR7).

### Construction of an Intrauterine Hypoxia Model

The pregnant rats in the hypoxia group were placed in a plexiglass cage from the 15th to the 21st day of gestation, and an oxygen analyzer (Pro OX120:BioSpherix, New York, NY) adjusted the nitrogen gas and air mixture flow and real-time monitoring of oxygen percentage in the organic glass cage, keeping the oxygen concentration at (10 + 1) %, which lasted for 4 h per day; at the same time, we added calcium chloride in the organic glass to absorb the excess carbon dioxide and water vapor in order to control the humidity, avoiding carbon dioxide retention, and acidosis. Before exposure to hypoxia, pregnant rats were intubated in the femoral artery with a catheter consisting of an arterial catheter, a connecting tube, and a three-way valve. After successful intubation, the incision was covered with gauze, and the rats were exposed to hypoxia. During hypoxia, blood was collected every hour by adjusting the three-way valve to connect it with a syringe, and the collected blood was immediately added to a blood gas detection chip (EG7+:Abbott, Lake Bluff, IL). The chip was inserted into a portable blood gas analyzer (I-STAT 200:Abbott), Oxygen partial pressure, blood oxygen saturation, and pH were measured. The arterial partial pressure of oxygen of pregnant rats was maintained at 50–55 mmHg, and the blood oxygen saturation was maintained at 80–85% (22–24). The pregnant rats in the control group were kept at room temperature with an oxygen concentration of 21% until natural delivery.

### Sample Collection

A total of 18 intestinal samples were collected from newborn rats throughout the study, including four from the first-day of the intrauterine hypoxia group (INH1), five from the seventh-day of the intrauterine hypoxia group (INH7), four from the first-day of normal group (NOR1), and five from the seventh-day of normal group (NOR7). DNA was extracted from all 18 samples and sequenced successfully for analysis in the bioinformatics analysis program. Four newborn rats in the experimental group and control group were randomly selected, within 4 h after birth (with no feeding) and euthanized by the decapitation method. In a sterile environment, the colon content samples of newborn rats were removed with a sterile scalpel and washed with sterile distilled water. The intestinal contents were dug out with a sterile scalpel for preservation in a freezer storage tube and immediately frozen at −80°C until further analysis. The remaining rats in both groups were fed their mothers' milk until the seventh day, and the intestinal contents were obtained by the same method.

### DNA Extraction and PCR Amplification

Microbial DNA was extracted from the colon contents of newborn rats, and DNA quality was checked by 1% agarose gel electrophoresis. The V3–V4 regions of the bacterial ribosomal RNA gene were amplified, the specific primers with barcode were synthesized, the PCR products were recovered by excision from the gel using a gel recovery kit, eluted by Tris-HCL buffer, detected by 2% agarose electrophoresis, and denatured by sodium hydroxide; single-stranded DNA fragments were generated; and a Miseq library was constructed.

Double-end sequencing was carried out using a Miseq platform (Illumina, Inc., CA, USA). According to the overlap relation between PE reads, paired reads were merged into a sequence, and quality control and filtration were carried out for read quality and merging effects at the same time. Barcode and primer sequences at both ends of the sequence were used to distinguish samples to obtain effective sequences and correct sequence directions. Reads below 50 bp after quality control were filtered, and N-base reads were removed for data optimization.

### Bioinformatics Analysis

Based on 97% similarity of optimized sequences, operational taxonomic units (OTUs) of non-repetitive sequences (excluding single sequences) were clustered to obtain an OTU abundance table. The OTUs were flattened based on the minimum number of sequences in a sample (34,479), and the OTUs with the total number of sequences annotated to the bacterial domain level were ≥20 to ensure the accuracy of subsequent data analysis. Alpha diversity indexes (Chao, ACE, Shannon, Simpson, coverage) were calculated to evaluate the richness, diversity, and coverage of the sample microbial community and to analyze the difference between the indexes. Principal coordinate analysis (PCoA) based on the binary Jaccard distance algorithm and non-metric multidimensional scaling analysis (NMDS analysis) based on the binary Jaccard distance algorithm were used to study the similarity or difference of sample community compositions and check whether the difference between groups was significant. LEfSe software was used to estimate the impact of the abundance of each species on the discriminant effect by linear discriminant analysis (LDA). Significant differences in COG categories in the four groups were determined using LEfSe analysis.

### Statistical Analysis

Student's *t*-test was used to analyze the differences between alpha diversity indexes. The Adonis test was used to analyze the difference between groups based on PCoA results groups. An ANOSIM test was used to analyze the difference between groups based on NMDS. Continuous variables are described as the means ± standard deviations unless otherwise stated. The Wilcoxon rank-sum test was used for species difference analysis between groups, and differences were considered significant when *P* < 0.05.

## Results

### Intrauterine Hypoxia Decreased the Birth Weights of Neonatal Rats

**Figure 1:**
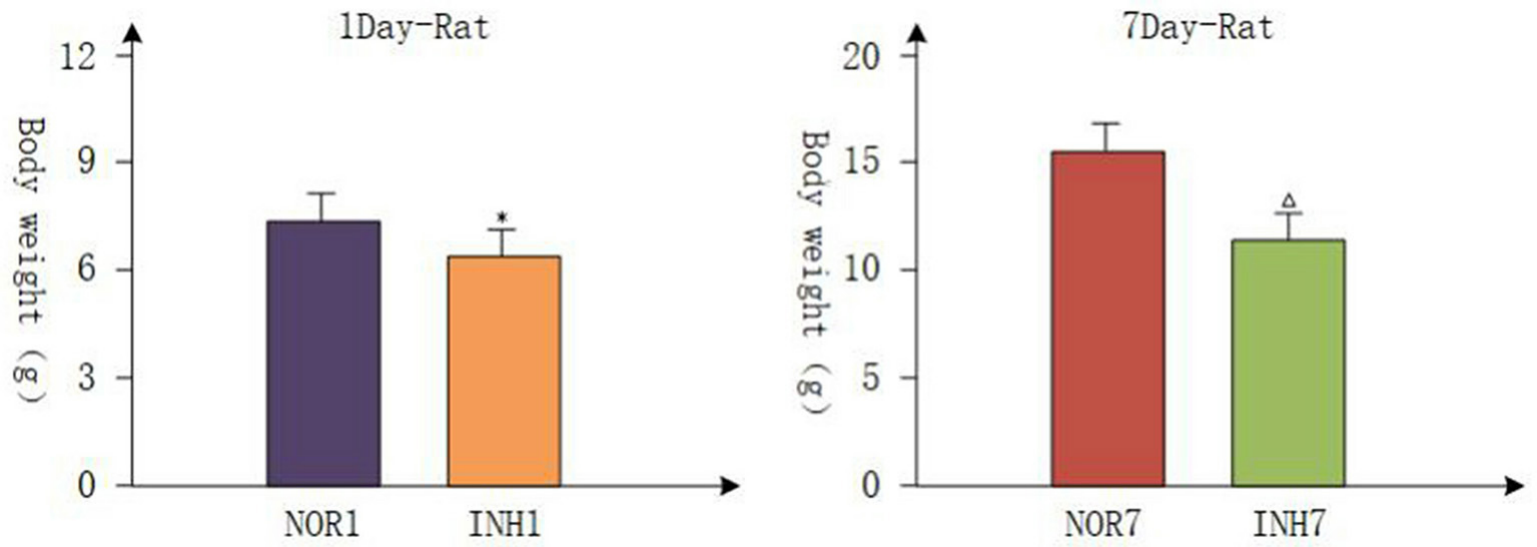
NOR1 vs. INH1: ^*^P < 0.05; NOR7 vs. INH7: Δ*P* < 0.05.

All pregnant rats gave birth naturally, and the body weights of newborn rats were measured within 2 h, which were accurate to 0.01 g. The mean gestation time of the hypoxia group and control group was 21.5 days (21–22 days), and the mean numbers of offspring were not different. Body weight loss is a common consequence of intrauterine hypoxia in newborns in the clinic (25, 26). Figure 1 shows the body weight data from each group. On the first day, the body weights of the INH1 (4 cases) and NOR1 (4 cases) groups were 6.380 ± 0.203 and 7.675 ± 0.263 g, respectively. On the seventh day, the body weights of the INH7 (*n* = 5) and NOR7 (*n* = 5) groups were 11.660 ± 0.615 and 15.460 ± 0.799 g, respectively. After intrauterine hypoxia intervention, rats in the hypoxia group weighed significantly less than rats in the normal group (*p* < 0.05) (Figure 1). These findings demonstrate that Wistar rats presented with typical characteristics of IUGR.

### Species Annotation and Evaluation

**Figure 2:**
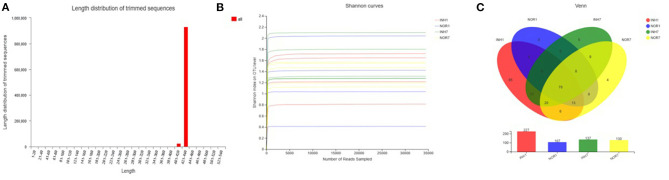
**(A)** Sequence length distribution diagram. The X-axis represents the length interval, and the Y-axis represents the number of sequences. **(B)** Alpha-diversity Shannon index dilution curve, with the horizontal axis representing the amount of randomly selected sequencing data and the vertical axis representing the number of observed species. **(C)** A Venn diagram that can be used to count the number of common and unique species in multiple groups of samples, which can intuitively show the similarity and overlap of species composition in different environmental samples. Different colors represent different groups, overlapping numbers represent the number of species common to multiple groups, and non-overlapping numbers represent the number of species unique to the corresponding group.

The total number of effective sequences and effective bases was 956,588 and 409,549,652 bp in the sequencing results, respectively. The average length of the sequences was 428.16. High-quality sequences with lengths in the range of 421–440 accounted for 97.28% of the total sequences. The mean of effective sequences of the intrauterine hypoxia group was 54,468, which was higher than the mean of 49,819 in the normal group (Figure 2A). The dilution curve of the alpha diversity index plateaued as the amount of data extracted increased, indicating that the sequencing data volume was reasonable (Figure 2B).

### OTU Analysis Results

A total of 17 phyla, 30 classes, 72 orders, 110 families, 181 genera, and 222 species were measured in the four groups, with a total of 253 OTUs. In the INH1 group, there were 17 phyla, 29 classes, 69 orders, 100 families, 152 genera, 183 species, and 198 OTUs. In the INH7 group, there were 5 phyla, 12 classes, 23 orders, 39 families, 48 genera, 59 species, and 69 OTUs. In the NOR1 group, there were 4 phyla, 8 classes, 16 orders, 22 families, 26 genera, 34 species, and 39 OTUs. In the NOR7 group, there were 4 phyla, 8 classes, 17 orders, 29 families, 39 genera, 49 species, and 56 OTUs. The number of species in each classification level in the hypoxia groups was higher than that in the control groups. Among them, there were 85 unique OTUs in the INH1 group, 5 unique OTUs in the INH7 group, 0 unique OTUs in the NOR1 group, and 4 unique OTUs in the NOR7 group (Figure 2C).

### Alpha Diversity Analysis

**Figure 3:**
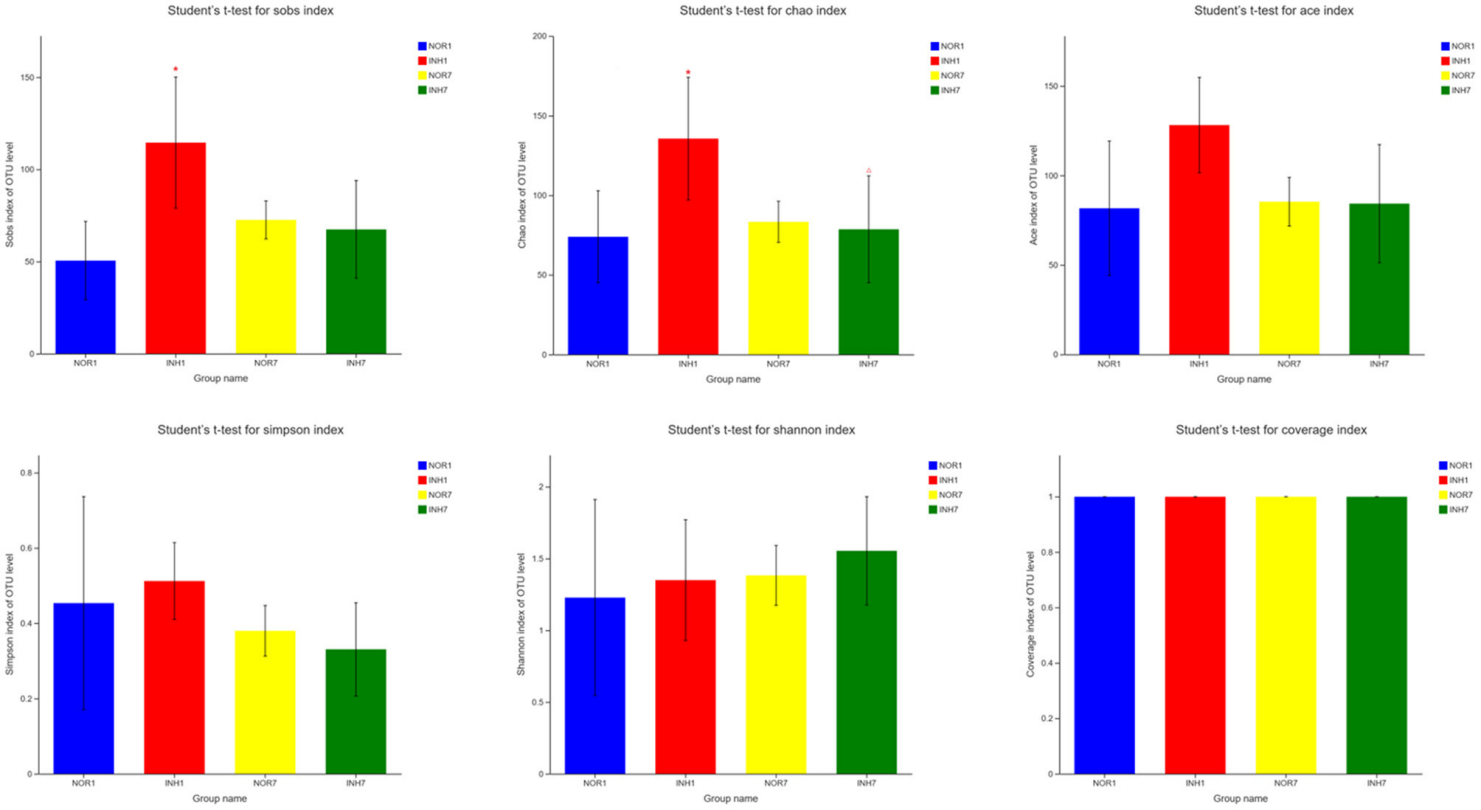
Alpha diversity index test results. NOR1 vs. INH1: ^*^P < 0.05; INH1 vs. INH7: Δ*P* < 0.05.

The mean values of the sobs, ACE, and Chao1 indexes reflecting community richness in the INH1 group were higher than those in the NOR1 group. The differences in the sobs and Chao indexes were significant (*P* < 0.05), as confirmed by Student's *t*-test. The mean values of the Shannon and Simpson indexes reflecting community diversity in the INH1 group were slightly higher than those in the NOR1 group (*P* > 0.05). Compared with those in the NOR7 group, the mean values of the sobs, ACE, Chao, and Simpson indexes in the INH7 group were slightly lower, while the mean values of the Shannon index were higher. There was no significant difference among the five indexes (*P* > 0.05). The mean value of the Shannon index of the INH1 group was slightly lower than that of the INH7 group, while the mean values of other indexes were higher than those of the INH7 group, and the difference in the Chao index was statistically significant (*P* < 0.05). There was no significant difference between the NOR1 group and the NOR7 group. The coverage indexes of all samples reflecting community coverage were 100%, indicating that the sequencing results could reflect the true microbial situation (Figure 3).

### Analysis of Community Composition

**Figure 4:**
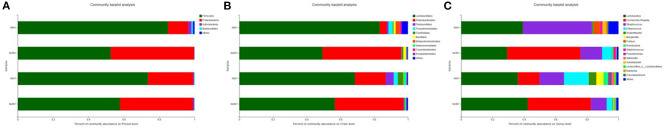
**(A)** Bar plot of phylum-level community. **(B)** Bar plot of order-level community. **(C)** Bar plot of genus-level community. The vertical axis represents the sample name, the horizontal axis represents the proportion of species in the sample, the columns with different colors represent different species, and the length of the columns represents the proportion of the species.

The dominant microbiota constituents of the four samples at the phylum level were Firmicutes and Proteobacteria, followed by Actinomycetes and Bacteroidetes. The mean relative abundance of Firmicutes in the hypoxia groups was higher than that in the control groups, but the mean relative abundance of Proteobacteria was lower than that in the control groups. The difference was not statistically significant (*P* > 0.05) (Figure 4A). At the order level, the dominant groups were Lactobacillales and Enterobacteriales. In the INH1 group, the abundance of Lactobacillales was significantly higher than that in NOR1, and the average relative abundance of Enterobacteriales was also significantly increased in the NOR1 group. In the INH7 group, the abundance of Enterobacteriales was lower than that in the INH1 group (Figure 4B). At the genus level, the dominant groups were Lactobacillus, Escherichia-Shigella, Streptococcus, and Enterococcus. In the INH1 group, the average relative abundances of Streptococcus and Lactobacillus were relatively high, and the abundances of Escherichia-Shigella and Enterococcus were significantly lower than those in the NOR1 group. In the INH7 group, the abundance of Escherichia-Shigella was lower than that in NOR7 but significantly increased compared with that in the INH1 group, and the average relative abundance of Enterococcus was also significantly increased compared with that in the INH1 group; its relative abundance was also higher than that in the NOR7 group (Figure 4C).

### Analysis of Beta Diversity

**Figure 5:**
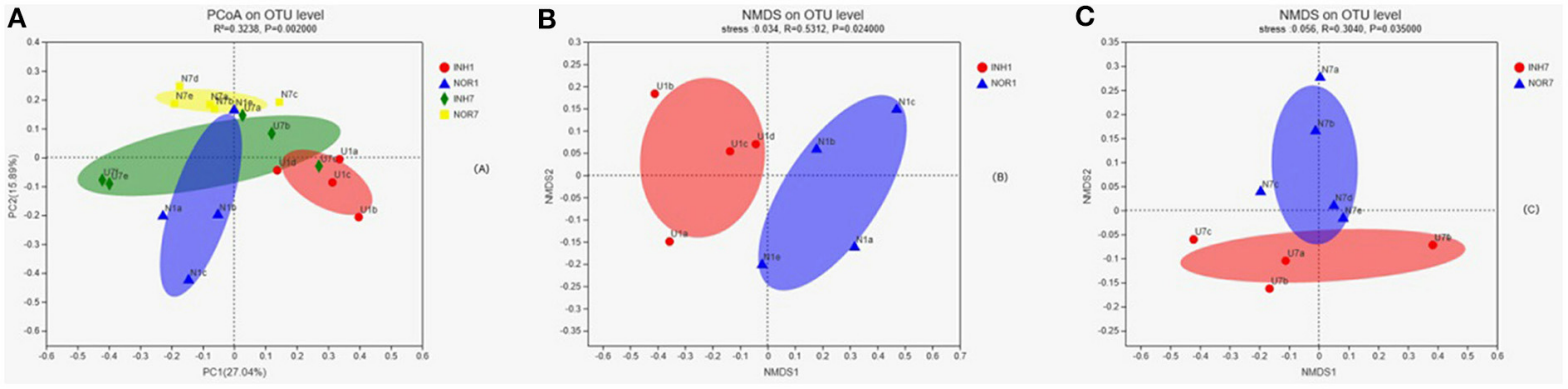
**(A)** In PCoA, the X-axis and Y-axis represent the two selected principal coordinate axes, and the percentage represents the explanatory value of the principal coordinate axes to the difference in sample composition; the X-axis and Y-axis scale is a relative distance, with no practical significance. Points of different colors or shapes represent samples of different groups. The closer the two sample points are, the more similar the species composition of the two samples is. Panels **(B,C)** the results of NMDS analysis. Points with different colors or shapes represent samples of different groups. The closer the two sample points are, the more similar the species composition of the two samples is. Horizontal and vertical coordinates represent relative distances and have no practical significance. Stress is based on a test of the strengths and weaknesses of NMDS analysis results. It is generally believed that stress < 0.2 can be represented by a 2d NMDS point graph, which has certain explanatory significance. When stress < 0.1, it can be considered a good ranking; when stress < 0.05, it is well-represented.

Principal coordinate7 analysis was performed on the four groups of samples based on the binary Jaccard distance algorithm, namely, PCoA, to explore the similarity and difference in the community composition of the four groups. An Adonis test was used to examine the difference between groups. The results showed that the difference in the community composition of the four groups was greater than the difference within the group (*R*^2^ = 0.3238), and the difference was statistically significant (*P* < 0.05) (Figure 5A). The INH1, NOR1, INH7, and NOR7 groups were subjected to NMDS analysis, namely, NMDS analysis, based on the binary Jaccard distance algorithm. ANOSIM was used for intergroup differences, showing that the differences in bacterial communities between groups were significant (*P* < 0.05) (Figures 5B,C).

### Species Difference Analysis

**Figure 6:**
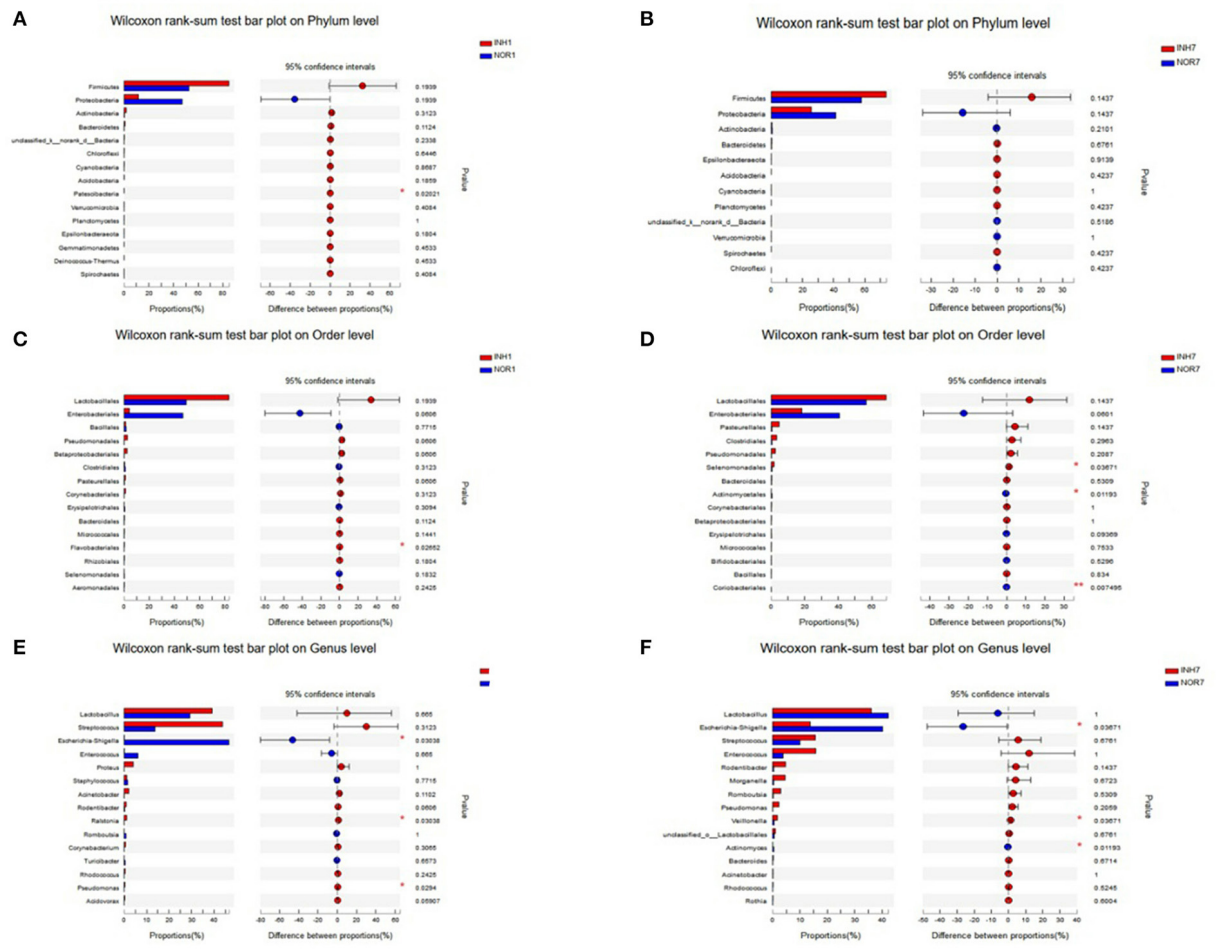
The X-axis represents different groups, different colored boxes represent different groups, and the Y-axis represents the average relative abundance of a species in different groups. **(A,B)** Bar plot on Phylum level in different groups. **(C,D)** Bar plot on Order level in different groups. **(E,F)** Bar plot on Genus level in different groups.

The Wilcoxon rank-sum test was used to detect significant differences between groups at the phylum, order, and genus levels for the four groups. At the phylum level, intrauterine hypoxia modified the proportions of the two principal phyla (Proteobacteria and Firmicutes) of the gut microbiota with respect to those in the normal group, and the percentage of Firmicutes in the INH1 group reached 84.95%. The average relative abundance of Firmicutes in the two intrauterine hypoxia groups was higher than that in the control group. The dominant microbiota constituent of the INH1 group was Firmicutes, and the dominant microbiota constituent of the NOR1 group was Proteobacteria. Regarding the low-abundance phylum Patescibacteria, the INH1 and NOR1 groups exhibited a significant difference (Figure 6A). There was no statistically significant difference between the INH7 group and the NOR7 group (Figure 6B). The relative abundances of Firmicutes and Bacteroidetes and the ratio of Firmicutes to Bacteroidetes (F/B) of the INH7 group were higher than those of the INH1 group and higher than those of the NOR7 group. The ratio of Firmicutes to Bacteroidetes (F/B ratio) is a biomarker of dysbiosis (27). At the order level, the INH1 and NOR1 groups showed significantly different abundances of Flavobacteriales, which is a major taxonomic order observed in ambient water samples (Figure 6C). There were significant differences between the INH1 and INH7 groups in the abundances of three orders: Selenomonadales, Actinomycetales, and Coriobacteriales (Figure 6D). At the genus level, the dominant groups did not change: Lactobacillus, Escherichia-Shigella, Streptococcus, and Enterococcus. The INH1 and NOR1 groups showed significant differences in abundances of the genera Escherichia-Shigella, Ralstonia, and Pseudomonas (*p* < 0.05). In the NOR1 and NOR7 groups, Escherichia-Shigella showed clear enrichment (Figure 6E). The INH7 and NOR7 groups showed significant differences in the abundances of three genera: a high abundance of Escherichia-Shigella and a relatively low abundance of Veillonella and Actinomyces (Figure 6F). Streptococcus and Veillonella often coexist in the intestinal ecosystem. We noted that the Escherichia-Shigella abundance markedly decreased and that the Streptococcus abundance increased in the hypoxia groups.

**Figure 7:**
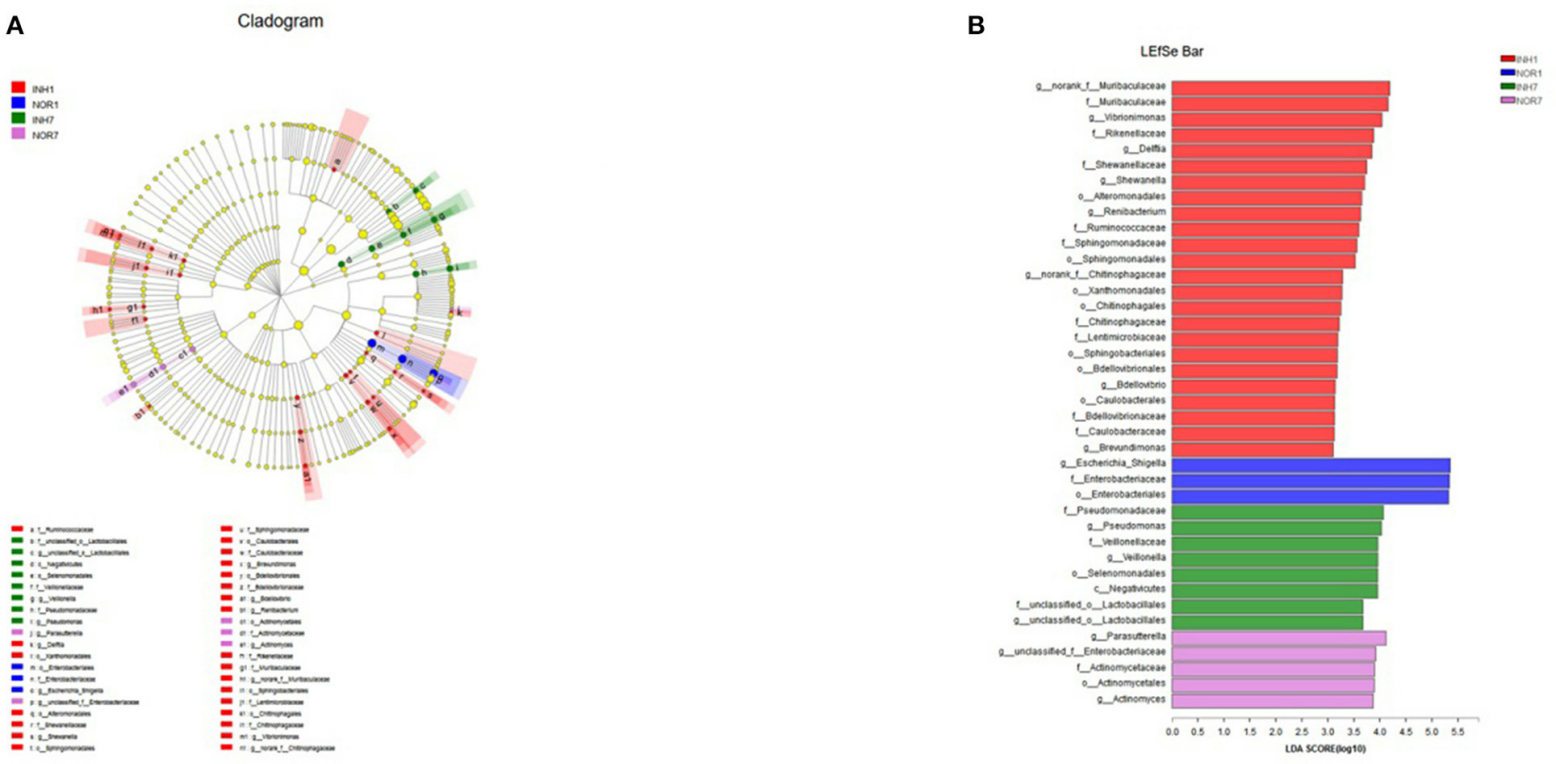
**(A)** Different color nodes represent the microbial groups that are enriched significantly in the corresponding groups and have a significant influence on the difference between the groups. The light yellow nodes represent the microbial groups that have no significant difference in different groups or have no significant influence on the difference between groups. **(B)** The LDA discriminant histogram shows the counts of the microbial communities with significant effects in multiple groups based on the LDA score obtained through LDA analysis (linear regression analysis). The larger the LDA score is, the greater the influence of species abundance on the different effects.

Next, we used LEfSe multistage species difference discriminant analysis to detect species with significant differences in abundance in the four groups of samples and used LDA to evaluate the impact of each species on different effects. The results showed statistically significant differences among the following species: for the INH1 group: Ruminobacteriaceae, Delft bacteria, Flavobacteriales, Alteromonadales, Shewanellaceae, Muribaculaceae, etc.; for the INH7: unclassified Lactobacillales, Negativicutes, and Pseudomonadaceae; and for the NOR1 group: Enterobacteriales, Escherichia-Shigella. NOR7: Parasutterella, unclassified-Enterobacteriaceae, and Actinomycetales (Figure 7A). In the INH1 group, the enrichment of Muribaculaceae had the greatest impact on the difference between groups. Furthermore, Pseudomonadaceae had the most significant influence on the INH7 group. LEfSe analysis showed an increased distribution of Escherichia-Shigella in the NOR1 group. In the NOR7 group, Parasutterella and unclassified Enterobacteriaceae had the most significant influence on the difference between groups. Microbial community structures have not yet been well-grouped according to the different etiologies (Figure 7B).

### COG Annotation and Analysis

**Figure 8:**
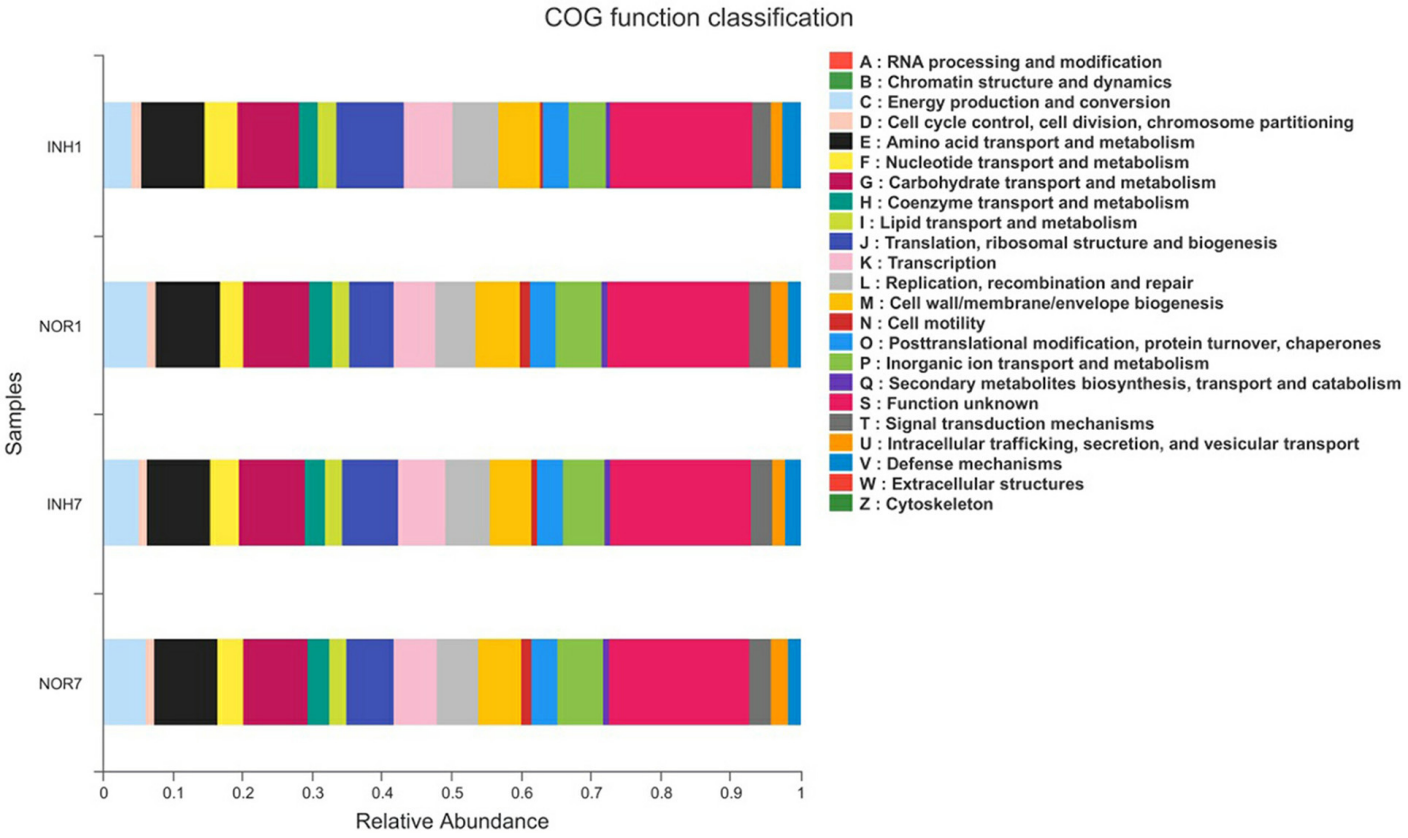
The X-axis represents the average relative abundance of a function in different groups, and different colors represent different functions. The Y-axis represents different groups.

We obtained COG family information through PICRUSt analysis to identify biologically significant differences in the four groups. The analysis provided insights into the functional properties of the gut microbiota. As shown in Figure 8, we found 23 different functional COG categories in the four groups. Of these categories, five functional COG categories were highly enriched in the NOR1 group, including unknown function [S], carbohydrate transport and metabolism [G], amino acid transport and metabolism [E], energy production and conversion [C], and translation, ribosomal structure, and biogenesis [J]. In contrast, the metagenomes of the INH1 group animals were enriched in unknown function [S]; translation, ribosomal structure, and biogenesis [J]; amino acid transport and metabolism [E]; carbohydrate transport and metabolism [G]; and transcription [K] (Figure 8). The metagenomes of the NOR7 group animals were enriched in unknown function [S]; carbohydrate transport and metabolism [G]; amino acid transport and metabolism [E]; translation, ribosomal structure, and biogenesis [J]; and inorganic ion transport and metabolism [P]. In the INH7 group, the enriched functional categories included unknown function [S]; carbohydrate transport and metabolism [G]; amino acid transport and metabolism [E]; translation, ribosomal structure, and biogenesis [J]; and transcription [K] (Figure 8). Notably, we found no significant difference in functional COG categories between the hypoxia group and the normal group.

## Discussion

Our results showed increased species richness and diversity of the gut microbiota on the INH1 in rats, and we identified several taxa that showed a significant difference in abundance between the hypoxia and control groups using *t*-tests and LEfSe analysis. These bacteria are closely linked to intestinal diseases.

The results show that no feeding newborn rats within 4 h after birth already contain diverse microbial communities, which is consistent with the latest viewpoint that initial microbiota colonization in the fetus before birth has already started (28, 29).

It is currently believed that an imbalance of the gut microbiota is associated with the occurrence of NEC, septicemia, bronchopulmonary dysplasia, and neurodevelopmental disorders (18, 19, 30, 31). Alpha diversity analysis was performed on the sequencing results for the gut microbiota of intrauterine hypoxia newborn rats, and the results showed that the diversity and richness of the gut microbiota in the first day of the intrauterine hypoxia group were higher than those of the control group, and the difference in richness was statistically significant. We can determine the reasons for this result from two aspects. First, pregnant rats were once fed milk containing Enterococcus carrying a gene marker, and Enterococcus containing the same gene marker was detected in fetal stool samples of their offspring (32), indicating that the gut microbiota of mothers is one of the potential sources of the gut microbiota of newborns. In our study, the pregnant rats after hypoxia induction may have produced blood by the “diving reflex,” diverting blood from the unimportant organs such as the bowel to vital organs (33), leading to intestinal mucosal damage and permeability enhancement and damaging the normal intestinal barrier protection function; intestinal bacteria are more likely to prompt matrix deposition through the intestinal epithelium, which involved in the vertical transfer of microorganisms in the womb, eventually transferring them to the newborn baby rat intestine. Thus, in the intestinal contents of the offspring, the observed abundance and diversity of the flora was higher than that in the control group animals. Additionally, studies have suggested that the neonatal gut microbiota is associated with swallowing amniotic fluid during fetal life (34, 35). A study on microbial molecular analysis of the amniotic fluid of premature infants found that the concentration of bacterial rDNA in the amniotic fluid was negatively correlated with the gestational age at delivery (36). Since gestational age is the best indicator to measure maturity (33), according to the results of the study, it can be speculated that the microbial community richness of the amniotic fluid is negatively correlated with fetal maturity, which could explain why on the first day of intrauterine hypoxia in this experimental group of newborn rats, the gut microbiota richness of the contents was higher than that in the control group because a change in richness is caused by a lack of oxygen with premature birth, as the system development is not mature.

Another interesting finding of this study was that after 7 days of suckling, the differences in the abundance and diversity of the INH7 and NOR7 group microbiota were not as significant as the differences between the two groups on the first day. The abundance of Streptococcus in the INH7 group was significantly lower than that in the INH1 group but still higher than that in the NOR7 group, while the abundance of Enterococcus and Escherichia increased significantly, and the common proportion of the three was close to that of Streptococcus in the INH1 group. Therefore, it can be inferred that there may be a competitive relationship among Streptococcus, Enterococcus, and Escherichia. The abundance and diversity of the microflora of neonatal rats with intrauterine hypoxia were closer to those of normal neonatal rats after suckling. We hypothesized that this was mainly due to a decrease in pathogenic bacteria with a higher abundance, which is consistent with previous studies showing that suckling can deplete harmful bacteria and enrich beneficial bacteria with a lower abundance. Suckling not only contributes to the formation of neonatal immune defense and reduces the colonization of pathogenic microorganisms but also induces the colonization of symbiotic organisms, thereby regulating intestinal inflammation to reduce intestinal damage and the risk of NEC (6, 37, 38). Breastfeeding is the most effective strategy to protect infants against NEC (6). Human milk oligosaccharides are non-digestible carbohydrates that are thought to influence the gastrointestinal microbiome and possibly protect against NEC (39, 40). In a single-center feeding study with a large sample size of 464 patients, the incidence of NEC in ELBW infants was found to be 1%, with only two deaths. This result was strongly associated with widespread breastfeeding and adherence to standardized feeding strategies (41). A retrospective study of breastfeeding in 550 very-low-birth-weight infants in the United States showed that continued breastfeeding significantly reduced the incidence of NEC in preterm infants (42).

Metagenomic sequencing has been widely applied in the comprehensive analysis of the relationships between microbial function and host physiology. We observed dynamic shifts in the compositions and functions of gut microbes and fecal metabolites in rats. The abundance of the INH1 group was higher than that of the NOR1 group in four functions, including chromatin structure and dynamics [B]; defense mechanisms [V]; cell motility [N]; and cytoskeleton [Z]. The data indicate that these molecular functions and biological processes might be implicated in the progression of various intrauterine hypoxia-induced complications. After 7 days, the functional differences between the two groups were narrowed, and we speculated that the gut microbiota of the rats after feeding tended to be consistent due to the influence of the diet and environment.

The results of species composition analysis showed that in the four groups of intestinal content samples, at the phylum level, the dominant gut microbiota constituents were Firmicutes and Proteobacteria, followed by Actinomycetes and Bacteroidetes, which was consistent with previous research results (43, 44). The mean relative abundance of Firmicutes in the two intrauterine hypoxia groups was higher than that in the two control groups, and the abundance proportion of Firmicutes in the INH1 group, in particular, reached 84.95%. The abundance of Proteobacteria in the NOR1 group was significantly higher than that in the INH1 group, and the ratio of Firmicutes to Bacteroidetes in the INH1 group was lower than that in the NOR1 group. After 7 days of dynamic observation, the gap between Firmicutes and Bacteroidetes abundances in the INH7 and NOR7 groups was narrowed, and the ratio of Firmicutes to Bacteroidetes in the INH7 group was higher than that in the INH1 group and that in the NOR7 group. Relevant studies have found that the ratio of Firmicutes to Bacteroidetes can affect the production of short-chain fatty acids. When the ratio increases, it can lead to a decrease in butyrate concentration in the body, promote the body's inflammatory response and destroy the integrity of the intestinal epithelial barrier, thus triggering an autoimmune response (45). Studies on inflammatory bowel disease, metabolic syndrome, and autism have found that the ratio of Firmicutes to Bacteroidetes has increased (46–48), suggesting that neonates with intrauterine hypoxia have an increased risk of these diseases in childhood and adulthood. At the genus level, the INH1 group microbiota was mainly composed of Streptococcus and Lactobacillus, among which a high proportion of Streptococcus may be one of the reasons for the susceptibility of neonates with intrauterine hypoxia to infection. The top three dominant strains of the NOR1 group were Escherichia, Lactobacillus, and Streptococcus, among which there were five species of the Escherichia genus. *Escherichia coli*, one of the common normal bacteria in the human intestinal tract, is involved in the synthesis of vitamin B and vitamin K. It is normally not pathogenic. Under certain conditions, some serotypes can cause gastrointestinal symptoms such as diarrhea and occasionally can also cause intestinal symptoms such as urinary tract infection. *Escherichia coli* is an important contributor to the initial colonization of the gut microbiota in normal newborns and plays an important role in the later colonization of Bifidobacteria. Additionally, it has been found that oral non-pathogenic *E. coli* strains (O83 and Nissle 1917) can induce local mucosal and systemic immunity, resulting in increased specific antibody levels detected in neonatal feces, saliva, and serum (49) and reducing the possibility of neonatal infection. Therefore, the low immune function of neonates with intrauterine hypoxia may be related to the lack of *E. coli* in the initial colonizing community. In the INH1 group, the abundance of gram-negative bacilli, such as Acinetobacter, Ralstonia, Pseudomonas, and Proteus, was higher than that in the NOR1 group. This enrichment may lead to injury of intestinal epithelial cells and increased mucosal permeability, and a large amount of lipopolysaccharide (LPS) can subsequently enter the blood to activate TLR4, which leads to the emergence of NEC (50, 51). Intestinal hemodynamic disorder caused by intrauterine hypoxia is the main pathophysiological factor that leads to NEC. Current studies have shown that perinatal hypoxia and ischemia processes and inflammation are the origin points of fetal intestinal injury causing NEC, and “hypoxic-ischemic enterocolitis (HIEnt)” has been proposed to define and distinguish this unique clinical entity (52). These opportunistic pathogens also increase the risk of sepsis in newborns (53, 54). After a 7-day observation period of suckling, the abundance of Streptococcus in the INH7 group was significantly lower than that in the INH1 group but still higher than that in the NOR7 group, while that of Enterococcus and *E. coli* was significantly increased, and the proportion of the three was similar to that of Streptococcus in the INH1 group. Therefore, it can be speculated that there may be a competitive relationship among Streptococcus, Enterococcus, and *E. coli*. Lactobacillus was the dominant gut microbiota constituent in the four groups of samples, further proving that vertical transmission of the maternal vaginal microbiota is one of the main sources of initial colonization of the neonatal gut microbiota (55).

In conclusion, intrauterine hypoxia affects the initial colonization of the gut microbiota in neonates to some extent, and neonates with intrauterine hypoxia have a higher risk of septicemia, NEC, infectious diseases, autoimmune diseases, inflammatory bowel disease, metabolic syndrome, and autism in childhood and adulthood than normal neonates. Although the microbial community of the INH7 group was significantly different from that of the INH1 group after suckling, it was still significantly different from that of the NOR7 group regarding the genera Escherichia-Shigella, Veillonella, and Actinomyces. In addition, the observation period of suckling in the present study was only 7 days, so the possibility of long-term influence cannot be ruled out.

In our study, the effect of intrauterine hypoxia on the intestinal microecology of neonatal rats in the early stage was investigated through high-throughput sequencing technology and a variety of bioinformatics analyses, providing a new idea for understanding the pathogenesis of related diseases of neonates with intrauterine hypoxia at birth, childhood, and adulthood. After abnormal colonization and proliferation of intestinal bacteria, the microbiota constituents participate in the pathophysiological process of NEC by generating metabolites and bacterial components and inducing an immune response. However, the specific mechanism by which gut microbiota dysregulation affects the development of NEC is still unclear and requires further research and exploration. The study of gut microbiota disorders provides a new idea for the early diagnosis of NEC, and microbiome optimization and correction of disorders provide a new strategy for the early prevention and treatment of NEC. As factors such as breastfeeding and intrauterine hypoxia are associated with NEC, further studies are needed to uncover the true contribution of these factors to microbiota development. Microbial dysbiosis preceding NEC in preterm infants is characterized by increased relative abundances of Proteobacteria and decreased relative abundances of Firmicutes and Bacteroidetes (56). Microbiome optimization may provide a novel strategy for preventing NEC.

Disturbances in microbiota development have been related to the development of disorders in the adult period. Our data indicate that intrauterine hypoxia could greatly influence health throughout life by interfering with gut microbiota development. In summary, intrauterine hypoxia may increase the risk of NEC by changing the colonization of the gut microbiota at birth. Babies born with intrauterine hypoxia require a more cautious feeding strategy and monitoring of abdominal conditions.

## Data Availability Statement

The data presented in the study are deposited in the SRA datebase repository, accession number (PRJNA706401).

## Ethics Statement

The animal study was reviewed and approved by Local ethics review Committee of Harbin Medical University.

## Author Contributions

CJ designed the study. YS, LL, JS, WM, and KX performed the experiments and analyzed the data. YS and LL wrote the manuscript. All authors contributed to the article and approved the submitted version.

## Conflict of Interest

The authors declare that the research was conducted in the absence of any commercial or financial relationships that could be construed as a potential conflict of interest.
